# The Changing Nature of Palliative Care: Implications for Allied Health Professionals’ Educational and Training Needs

**DOI:** 10.3390/healthcare7040112

**Published:** 2019-09-28

**Authors:** Deidre D. Morgan, Deb Rawlings, Carly J. Moores, Lizzie Button, Jennifer J. Tieman

**Affiliations:** 1 Palliative and Supportive Services, College of Nursing and Health Sciences, Flinders University, Adelaide 5001, Australia; deborah.rawlings@flinders.edu.au (D.R.); jennifer.tieman@flinders.edu.au (J.J.T.); 2 CareSearch, Flinders University, Adelaide 5001, Australia; 3 Nutrition and Dietetics, College of Nursing and Health Sciences, Flinders University, Adelaide 5001, Australia; carly.moores@flinders.edu.au

**Keywords:** allied health, education/training, palliative care, education

## Abstract

CareSearch is an Australian Government Department of Health funded repository of evidence-based palliative care information and resources. The CareSearch Allied Health Hub was developed in 2013 to support all allied health professionals working with palliative care clients in all clinical settings. This cross-sectional online survey sought to elicit allied health professionals palliative care experiences and subsequent considerations for educational and clinical practice needs. The survey was disseminated nationally via a range of organisations. Data was collected about palliative care knowledge, experience working with palliative care clients and professional development needs. Data were evaluated by profession, experience and practice setting. In total, 217 respondents answered one or more survey questions (94%). Respondents (65%) reported seeing >15 palliative care clients per month with 84% seen in hospital and community settings. Undergraduate education underprepared or partially prepared allied health professionals to work with these clients (96%) and 67% identified the need for further education. Access to postgraduate professional development was limited by available backfill and funding. Study findings support the importance of free, accessible, relevant educational and professional development resources to support clinical practice. This is particularly relevant for allied health professionals who have limited opportunities to attend formal professional development sessions.

## 1. Introduction

The World Health Assembly Resolution (2014) recommends palliative care be considered as a component of comprehensive care throughout the life course due to rapidly increasing numbers of people with palliative care needs [1]. However, achieving this requires a whole-of-workforce approach and appropriately skilled professionals. Palliative care is provided by generalist and specialist services within primary and community sectors and by a range of health professionals in different settings, individually and as part of multidisciplinary teams. In Australia, allied health professionals comprise around 20% of the health workforce and deliver 200 million services per year, although it is worth noting that rural and remote populations find it difficult to access allied health professionals [2].

Allied health professionals play a key role in the care of people with palliative care needs in all health care settings (acute, subacute, ambulatory and community care). They bring profession-specific knowledge and skills that can be essential in addressing the person’s individual care needs. They can provide non-pharmacological symptom control options alongside pharmacological interventions. Their care approaches enable continuing participation in essential and valued daily activities [3,4]. Allied health interventions do not focus on curative care, rather they focus on optimising function for as long as possible and on identification of patient priorities at this time of their life. Importantly, allied health interventions, both physical and psychological, serve to mediate adjustment to functional decline and deterioration at the end-of-life. Interventions will often be delivered within the context of a multidisciplinary approach, even if the allied health professional works as a sole practitioner. 

Allied health professionals can make important contributions in the treatment and non-pharmacological management of symptoms, alongside improving the quality of life for palliative care patients and caregivers. Increasing numbers of allied health professionals employ a rehabilitative approach to optimise function of palliative care patients [4]. Management of symptoms such as dyspnea and fatigue to support comfort and function is an integral part of physiotherapy and occupational therapy interventions [4,5,6,7,8]. Psychosocial needs for patients and families around grief, loss and bereavement may be addressed by psychologists and social workers [9,10,11], while speech pathologists address communication, cognition and swallowing difficulties to support dignity, safety and comfort [12,13].

Little research considers the type of end-of-life care interventions being provided outside of specialist palliative care settings, the experiences of allied health professionals and their education and practice needs in these non-specialist settings. The purpose of this study was to survey allied health professionals across Australia on their understanding and views about palliative care, of its relevance to practice, their knowledge, and their clinical information education needs. The aim of this paper is to present survey results pertaining to palliative care training and continuing educational needs. Qualitative survey results presenting allied health professionals’ knowledge and views about palliative care are presented elsewhere [14].

## 2. Materials and Methods 

This study was a purpose-designed cross-sectional survey of Australian Allied Health Professionals. For the purposes of this survey, allied health professionals included were those most likely to work with palliative care clients in specialist or generalist settings. Survey functionality and face validity was determined by piloting the questions with the Executive Officer of Allied Health Professions Australia (AHPA) and representatives of the CareSearch Allied Health Hub advisory group prior to distribution (available from authors on request). The survey design and conduct were reviewed against the CHERRIES checklist [15]. 

### 2.1. Study Sample and Design

Ethics approval for this study was provided by the Flinders University Social and Behavioural Research Ethics Committee (Project 7014). Participant consent was implied through commencement of the online questionnaire. 

### 2.2. Recruitment

Allied health professionals were recruited to complete the survey through member newsletters of the Allied Health Professions of Australia and through social media channels such as CareSearch, LinkedIn and Twitter. Allied health professionals approached to participate in the survey included occupational therapists, physiotherapists, speech pathologists, social workers, dietitians, psychologists and music therapists. These allied health disciplines were represented in the CareSearch Allied Health Hub. It is not possible to determine how many people were offered the opportunity to participate in the survey due to the variety of approaches via member networks and social media but it is estimated that the networks alone represented more than 120,000 allied health professionals.

### 2.3. Data Collection

Participants accessed the participant information sheet and the online survey through embedded hyperlinks within newsletters or social media posts. Data were collected from 1 November 2015 to 30 April 2016, and included 42 items on palliative care education and professional development, practice, and attitude.

### 2.4. Data Analysis

This paper reports on demographic data and descriptive statistics. Categorical data are reported as number of respondents (*n*) and proportion (%). As it was not compulsory to respond to each survey item, there are varying levels of missing data for every question. Individual *n* values are presented for each item to indicate completeness of responses. Differences in proportions between groups were evaluated using Chi-square statistics. Statistical analyses were conducted using IBM SPSS version 23.0 (IBM Corp) and statistically significant differences (*p* < 0.05) are highlighted in bold.

## 3. Results

Two hundred and seventeen respondents answered 1 or more questions in the survey; of these, 49 responses (23%) were incomplete. Sociodemographic characteristics are reported in Table 1. The majority of respondents were female (94%) and young to middle aged. More than half of respondents were from the Eastern states of Australia.

### 3.1. Allied Health Professional Practice and Setting 

Occupational therapists, social workers, physiotherapists and dietitians comprised 86% of the sample (Table 1). Fifty seven percent of survey respondents had been practising for more than 11 years and 25% had been practicing for 6–10 years. More than half of the respondents were working in a hospital setting (52%), and approximately a third were working in specialist palliative care services or hospices (33%) and community settings (32%). Thirty-two percent of respondents practiced in more than one setting.

### 3.2. Palliative Care Training and Continuing Educational Needs of Allied Health Professionals

Over half of allied health professionals felt their undergraduate training did not prepare (54%), or only partially prepared them (42%) to care for palliative care patients and/or their families. Many allied health professionals had previously undertaken professional development in palliative care, most frequently by reading and short in-service training sessions within their workplace. Palliative care educational opportunities appeared to be frequent, with 68% reporting their most recent opportunity was within the last year. Over one-third (39%) had completed online learning modules. While approximately 10% had specific qualifications in palliative care, 67% indicated that they needed additional education to better inform their clinical practice when working with palliative care patients. However, respondents reported barriers to accessing education, most frequently citing time (41%) and funding (33%) as primary limitations. Lack of available leave or backfill to cover clinical caseloads and limited knowledge about specialised or relevant educational opportunities also prevented access to education (Table 1).

There were no significant differences in palliative care qualifications or need to access palliative care education by allied health discipline. However, speech pathologists (70%) and physiotherapists (75%) felt the least prepared by undergraduate education and reported needing higher levels of postgraduate training in order to work effectively with palliative care patients (data not shown).

Further training in grief and loss (56%) and symptom management (54%) were identified as priorities by the majority of disciplines (Table 2). However, there appeared to be differences between professions in regards to their palliative care education needs relating to pain and symptom management, and non-malignant disease. Speech pathologists were more likely to need training around managing grief and loss, dietitians around symptom management, and physiotherapists around care of people with non-malignant diseases. Those working in specialist palliative care services were less likely to need education around symptom management than those working in other clinical settings. Of note, education around grief and loss and palliative care needs of non-malignant populations was important to allied health professionals in all settings. Respondents with the least experience post-training reported needing further education in grief and loss (92%) compared to 56% of allied health professionals overall.

When asked to reflect on difficulties encountered in their most recent palliative care experience, most allied health professionals reported caseload or time demands as limiting (57%), while some reported feeling overwhelmed discussing non-medical topics (15%), and at their inability to take time out (14%). The proportion of allied health professionals who reported changing mindsets about working with palliative care patients differed by years of experience post-training. Those with the least experience reported this to be a greater problem at more than double the number of professionals overall (46% versus 23%). Additionally, the proportion citing a lack of palliative care knowledge or skills making their palliative care experiences difficult was significantly higher for those with less experience and those working outside of specialist palliative care settings. Forty-three percent of all respondents noted that access to postgraduate professional development was limited by available time, cost/funding, workload pressures and backfill. Further, allied health professionals working outside of a palliative care service were far more likely to report feeling overwhelmed discussing non-medical topics compared with medical topics than those working within this setting.

## 4. Discussion

A diverse allied health workforce delivers patient care in metropolitan, regional, rural, and remote settings. Each setting brings with it specific workforce issues and needs. Given lower life expectancies and high levels of chronic disease in lower socioeconomic areas, existing allied health models of care need to take into consideration ways to best support patients and caregivers at the end-of-life [16]. For example, Services for Australian Rural and Remote Allied Health (SARRAH) endorsed models of care that may address these needs include specialist outreach services, local community multi-disciplinary primary health teams, and augmented as needed but not replaced by telehealth [17].

A recent study of occupational groups working in long-term care settings highlighted the differences in palliative care-specific educational needs and the intensity of inter-professional collaboration [18]. Understanding the needs of different allied health disciplines contributes to workforce planning, enabling more targeted education and continuing professional development opportunities. It may be important to consider modes of educational delivery given the diversity of settings in which allied health professionals work and their involvement with chronic disease and with older Australians, many of whom will be approaching the end of the life in residential or community settings. Achieving provision of the best possible consumer-focused palliative care to patients and caregivers requires a systematic approach that supports allied health professionals working across all settings of care. Systems that do not address the needs of rural and remote allied health professionals who may feel overwhelmed, isolated and have unmet professional development needs, run the risk of reducing allied health professional retention [18]. Survey respondents identified unmet needs in all of these three areas, in particular around grief and loss and the evolving area of non-pharmacological symptom management. Those allied health professionals working outside specialist palliative care services as well as those with less clinical experience were more likely to be overwhelmed by the needs of patients at the end-of-life. This is of vital importance to any allied health professionals who may work in isolation or with limited access to peer support from other allied health professionals with specialist palliative care expertise.

Online resources targeting context-specific needs have been found to build confidence of allied health professionals who work with palliative care patients and were accessed by survey respondents. Australian palliative care resources that specifically address the allied health context such as the Allied Health section on the CareSearch website [19] and the End of Life Essentials online multidisciplinary modules [20] may be particularly important for the Australian allied health workforce, including those working in rural and remote settings [21]. While aimed at acute settings, the communication methods and principles in End-of-Life Essentials are relevant to clinicians working in all settings. The Program of Experience in the Palliative Approach (PEPA) also provides targeted placements (up to four days) for allied health professionals in order to develop palliative care clinical skills and an online learning module to support allied health professional development, workshops and support through clinical networking [22]. The PEPA program also provides paid backfill for health professionals that undertake a palliative care experiential learning placement.

This study had some limitations. Respondents were a self-selecting sample of allied health professionals, some of whom already worked in palliative care. This may have led to under-representation of generalist clinician professional development needs. Numbers of respondents are small and not equal for all disciplines, hence their responses may not be representative of individual discipline educational needs.

## 5. Conclusions

Allied health professionals have an active role to play in the physical, social, emotional and spiritual care of palliative care patients and their caregivers. However, in order to do this allied health professionals require access to evidence-based education to enable better support of patients and their caregivers as deterioration ensues. Allied health professionals working in rural and remote locations often take on specialist generalist roles but require adequate support to provide quality care to patients with complex palliative care needs. Generalist allied health professionals in particular require targeted post-graduate education, particularly around grief and loss and non-pharmacological symptom management. Online resources such as CareSearch and End-of-Life Essentials and clinical placements such as those offered through PEPA can support all allied health professionals in non-specialist palliative care settings to develop and sustain clinical skills through best available evidence and clinical networking. 

## Figures and Tables

**Table 1:** Sociodemographics of allied health professionals.

	*n*	%
**Sex**	*n* = 158	
Male	10	6
Female	148	94
**Age**	*n* = 160	
18–29 years	33	21
30–39 years	49	31
40–49 years	35	22
50–59 years	34	21
≥60 years	9	6
**State**	*n* = 144	
VIC	41	28
NSW	35	24
QLD	32	22
SA	19	13
WA	10	7
TAS	5	3
ACT	1	1
NT	1	1
**What is your profession?**	*n* = 160	
Occupational Therapist	45	28
Social Worker	35	22
Physiotherapist	28	18
Dietitian	26	16
Music Therapist	10	6
Speech Pathologist	10	6
Registered Nurse	3	2
Psychologist	2	1
More than one profession	1	1
**Years of experience post-training**	*n* = 160	
<2 years	13	8
2–5 years	16	10
6–10 years	40	25
11–20 years	41	26
>20 years	50	31
**Setting ^a^**	*n* = 146	
Hospital	76	52
Specialist palliative care service/hospice	48	33
Community	47	32
Residential Aged Care	15	10
Private Practice	11	8
Other	11	8

NB proportions may not add up to 100% due to rounding. ^a^ participants chose more than one response, i.e., responses are not mutually exclusive.

**Table 2:** Palliative care educational needs by discipline, years of experience and practice setting.

		Grief and Loss, *n* (%)	Symptom Management, *n (%)*	Palliative Care and Non-Malignant Disease, *n* (%)	Anxiety and Depression, *n* (%)	Communication, *n* (%)	Palliative Care and Cancer, *n* (%)	Pain Management, *n* (%)	Relationship Issues, *n* (%)
Total (*n* = 147)		83 (56)	79 (54)	73 (50)	71 (48)	65 (44)	64 (44)	63 (43)	56 (38)
Profession (*n* = 139)	Occupational Therapist (*n* = 41)	20 (49)	21 (51)	16 (39)	19 (46)	14 (34)	20 (49)	20 (49)	14 (34)
	Social Worker (*n* = 30)	15 (50)	8 (27)	14 (47)	17 (57)	13 (43)	10 (33)	9 (30)	17 (57)
	Physiotherapist (*n* = 25)	16 (64)	17 (68)	18 (72)	15 (60)	13 (52)	14 (56)	16 (64)	8 (32)
	Dietitian (*n* = 20)	13 (65)	16 (80)	8 (40)	8 (40)	11 (55)	11 (55)	4 (20)	5 (25)
	Music Therapist (*n* = 9)	4 (44)	4 (44)	3 (33)	2 (22)	3 (33)	2 (22%)	5 (56)	6 (67)
	Speech Pathologist (*n* = 10)	8 (80)	6 (60)	6 (60)	4 (40)	5 (50)	4 (40)	2 (20)	2 (20)
	Other (*n* = 4)	2 (50)	2 (50)	4 (100)	1 (25)	2 (50)	2 (50)	3 (75)	1 (25)
	*P* (χ^2^)	0.482	0.009	0.041	0.380	0.694	0.431	0.014	0.080
Years of experience post-training (*n* = 139)	<2 y (*n* = 12)	11 (92)	9 (75)	7 (58)	7 (58)	6 (50)	9 (75)	5 (42)	5 (42)
	2–5 y (*n* = 15)	11 (73)	10 (67)	10 (67)	11 (73)	10 (67)	9 (60)	6 (40)	7 (47)
	6–10 y (*n* = 37)	19 (51)	20 (54)	17 (46)	14 (38)	11 (30)	16 (43)	15 (41)	14 (38)
	11–20 y (*n* = 36)	15 (42)	15 (42)	12 (33)	13 (36)	15 (42)	12 (33)	15 (42)	11 (31)
	>20 y (*n* = 39)	22 (56)	20 (51)	22 (56)	21 (54)	19 (49)	16 (41)	18 (46)	16 (41)
	*P* (χ^2^)	0.023	0.251	0.146	0.076	0.147	0.091	0.988	0.819
Setting (*n* = 128)	PC/Hospice (*n* = 44)	20 (46)	12 (27)	24 (55)	20 (46)	13 (30)	13 (30)	18 (41)	15 (34)
	Other (*n* = 84)	49 (58)	56 (67)	41 (49)	40 (48)	39 (46)	46 (55)	36 (43)	32 (38)
	*P* (χ^2^)	0.193	<0.001	0.580	0.854	0.088	0.009	0.853	0.703

Data presented as *n* (%); χ^2^ test for proportion.

## References

[1] World Health Assembly Strengthening of Palliative Care as a Component of Comprehensive Care Throughout the Life Course. http://apps.who.int/medicinedocs/documents/s21454en/s21454en.pdf.

[2] Solomon D., Graves N., Catherwood J. (2015). Allied health growth: What we do not measure we cannot manage. Human Resources Health.

[3] Cobbe S., Kennedy N. (2012). Physical function in hospice patients and physiotherapy interventions: A profile of hospice physiotherapy. J. Palliat. Med..

[4] Barawid E., Covarrubias N., Tribuzio B., Liao S. (2015). The Benefits of Rehabilitation for Palliative Care Patients. Am. J. Hosp. Palliat. Care.

[5] Halkett G.K., Ciccarelli M., Keesing S., Aoun S. (2010). Occupational therapy in palliative care: Is it under-utilised in Western Australia?. Aust. Occup. Ther. J..

[6] Morgan D.D., White K.M. (2012). Occupational therapy interventions for breathlessness at the end of life. Curr. Opin. Support Palliat. Care.

[7] Occupational Therapy Australia (2015). Occupational Therapy in Palliative Care: Position Paper.

[8] Booth S. (2013). Cambridge Breathlessness Intervention Service (CBIS). Progress Palliat. Care.

[9] Kasl-Godley J.E., King D.A., Quill T.E. (2014). Opportunities for psychologists in palliative care: Working with patients and families across the disease continuum. Am. Psychol..

[10] Bosma H., Johnston M., Cadell S., Wainwright W., Abernethy N., Feron A., Kelley M.L., Nelson F. (2010). Creating social work competencies for practice in hospice palliative care. Palliat. Med..

[11] Payne M. (2009). Developments in end-of-life and palliative care social work: International issues. Int. Soc. Work.

[12] O’Reilly A.C., Walshe M. (2015). Perspectives on the role of the speech and language therapist in palliative care: An international survey. Palliat. Med..

[13] Pollens R.D. (2012). Integrating speech-language pathology services in palliative end-of-life care. Top. Lang. Disord..

[14] Morgan D.D., Rawlings D., Button E., Tieman J. (2019). Allied health clinicians’ understanding of palliative care as it relates to patients, caregivers and health practitioners: A cross sectional survey. J. Allied Health.

[15] Eysenbach G. (2004). Improving the quality of web surveys: The Checklist for Reporting Results of Internet E-Surveys (CHERRIES). J. Med. Internet Res..

[16] Services for Australian Rural and Remote Allied Health (SARRAH) (2016). Models of Allied Health Care in Rural and Remote Australia. Position Paper.

[17] Campbell N., McAllister L., Eley D. (2012). The influence of motivation in recruitment and retention of rural and remote allied health professionals: A literature review. Rural Remote Health.

[18] Kaasalainen S., Sussman T., Bui M., Akhtar-Danesh N., Laporte R.D., McCleary L., Wickson Griffiths A., Brazil K., Parker D., Dal Bello-Haas V. (2017). What are the differences among occupational groups related to their palliative care-specific educational needs and intensity of interprofessional collaboration in long-term care homes?. BMC Palliat. Care.

[19] CareSearch Allied Health and Palliative Care. https://www.caresearch.com.au/caresearch/tabid/2880/Default.aspx.

[20] End-of-Life Essentials. https://www.caresearch.com.au/caresearch/tabid/3699/Default.aspx.

[21] Ray R.A., Fried O., Lindsay D. (2014). Palliative care professional education via video conference builds confidence to deliver palliative care in rural and remote locations. BMC Health Serv. Res..

[22] PEPA Program of Experience in the Palliative Approach. https://pepaeducation.com/placements/health-professionals/allied-health-practitioners.

